# The Role of Neprilysin in Regulating the Hair Cycle

**DOI:** 10.1371/journal.pone.0055947

**Published:** 2013-02-13

**Authors:** Naoko Morisaki, Atsushi Ohuchi, Shigeru Moriwaki

**Affiliations:** Biological Science Laboratories, Kao Corporation, Haga-gun, Tochigi, Japan; University of Iowa, United States of America

## Abstract

In most mammals, each hair follicle undergoes a cyclic process of growing, regressing and resting phases (anagen, catagen, telogen, respectively) called the hair cycle. Various biological factors have been reported to regulate or to synchronize with the hair cycle. Some factors involved in the extracellular matrix, which is a major component of skin tissue, are also thought to regulate the hair cycle. We have focused on an enzyme that degrades elastin, which is associated with skin elasticity. Since our previous study identified skin fibroblast elastase as neprilysin (NEP), we examined the fluctuation of NEP enzyme activity and its expression during the synchronized hair cycle of rats. NEP activity in the skin was elevated at early anagen, and decreased during catagen to telogen. The expression of NEP mRNA and protein levels was modulated similarly. Immunostaining showed changes in NEP localization throughout the hair cycle, from the follicular epithelium during early anagen to the dermal papilla during catagen. To determine whether NEP plays an important role in regulating the hair cycle, we used a specific inhibitor of NEP (NPLT). NPLT was applied topically daily to the dorsal skin of C3H mice, which had been depilated in advance. Mice treated with NPLT had significantly suppressed hair growth. These data suggest that NEP plays an important role in regulating the hair cycle by its increased expression and activity in the follicular epithelium during early anagen.

## Introduction

As the hair cycle is one of the intrinsic and cyclic systems of regenerating tissue, the mechanism of its regulation is intriguing with respect to tissue reconstruction. There are many biological factors which have been reported to regulate or to synchronize with the hair cycle. Those factors can be divided into several classifications, such as hormones, growth factors, enzymes and transcription factors. Examples of enzymes include urokinase [1], ornithine decarboxylase [2], γ-glutamyl transpeptidase [3], alkaline phosphatase [4], hydroxysteroid dehydrogenase [5], adenyl-cyclase [6], aryl hydrocarbon hydroxylase [7], aromatase [8], glutathione s-transferase [9] and nexin 1, a serine protease inhibitor [10]. Since the hair cycle might be considered as a process of tissue regeneration, we thought that regulation of the extracellular matrix (ECM) could well affect the hair cycle. With regard to the ECM, proteoglycans have been well investigated and associated with the hair cycle [11]–[15]. However, only a few matrix degrading enzymes have been reported to be associated with the hair cycle so far, such as type IV collagenase [16], matrix metalloproteinase (MMP)-2 and TIMP-1 [1], [17]. Since we previously identified neprilysin (NEP) as dermal fibroblast elastase [18], we focused on the potential role of NEP in the regulation of the hair cycle.

Neprilysin is a cell surface metalloprotease, which is also known as neutral endopeptidase (NEP; EC 3.4.24.11), CD10, CALLA and enkephalinase. It is expressed in various tissues including the central and the peripheral nervous systems, normal and neoplastic lymphoid cells, and adrenal glands [19]. It is also expressed in normal skin such as eccrine glands and sebaceous glands, in cultured keratinocytes and fibroblasts [20], as well as in hair follicles and hair tumors [21]. NEP can degrade a wide variety of bioactive peptides, for example enkephalins [22], bradykinin [23], neurotensin, substance P [23], [24], CGRP [25], natriuretic peptide [26], fMet-Leu-Phe [27], endothelin [26], [28], and galanin [29]. We previously showed that NEP also has elastase activity and plays important roles during intrinsic and UV-induced skin aging [18]. We now report the role of NEP in regulating the hair cycle.

## Materials and Methods

### Animals

All procedures performed in this study were in accordance with instructions and permissions of the Institutional Animal Care and Use Committee of Kao Corporation. The Committee had approved this study and the Permit Numbers were 20071106-42 (rats) and 20071106-64 (mice).

Male Sprague-Dawley (SD) rats and male C3H mice were purchased from Charles River Japan Inc. (Yokohama, Japan). Animals were cared for in accordance with our Institutional Guidelines. The animals were fed ad libitum and were housed under conventional conditions at a controlled temperature (23±2°C), humidity (55±10%) and light (12 h light/12 h dark). For sampling skin biopsies from each stage of the hair cycle, we used rats 3 to 12 weeks at age (n = 5 at each week of age). We tried to reduce the number of animals used to a minimum to ameliorate animal welfare. After they reached a certain age, mice were euthanized with CO_2_ gas. We excised the dorsal skin of each rat from just below the line connecting both scapula to obtain specific hair cycle stage specimens, because all the dorsal skin would not be in the same phase. Each skin specimen was separated from the subcutaneous tissue and was sampled by punch biopsy to perform several assays (enzyme activity, protein expression, mRNA expression and immunohistochemistry).

For the enzyme activity assay, each skin biopsy was homogenized and solubilized in 0.1% Triton-X 100, 0.2 M Tris-HCl (pH 8.0) buffer, followed by ultrasonication and then by centrifugation (3000 rpm×20 min) to obtain supernatants for enzyme assays.

### Materials

The synthetic substrate for elastase, N-succinyl-tri-alanyl-p-nitroaniline (STANA) was purchased from Peptide Institute Inc (Osaka, Japan). Phosphoramidon was obtained from Boehringer Mannheim (Mannheim, Germany). Other chemicals, of reagent grade, were purchased from Sigma (St. Louis, MO, USA**)**.

### Measurement of Enzyme Activities

Elastase activity was measured as previously described [30]. In brief, the enzyme solution was added to the synthetic substrate STANA, and was incubated for 1 h at 37°C. The release of p-nitroaniline was measured by absorbance at 405 nm and enzymatic activities are expressed as units per mg protein, one unit representing the activity that releases 1 nmol of nitroaniline per h.

NEP activity was measured as previously described [31]. Enzyme solutions were diluted with MES buffer, and then the substrate solution (10 mM Glutaryl-Ala-Ala-Phe-4-methoxy-2-naphthylamine/dimethylformamide) was added and incubated for 1 h at 37°C to form Phe-4-methoxy-2-naphthylamine. The enzyme reaction was stopped by adding 400 µM phosphoramidon, after which 20 mU aminopeptidase M was added to form 4-methoxy-2-naphthylamine. The amounts of 4-methoxy-2-naphthylamine formed were measured using a spectrophotofluorometer at an excitation wavelength of 340 nm and an emission wavelength of 425 nm, and was used as the activity of NEP.

Type I and Type IV collagenase activities were measured using commercial assay kits (YAGAI corp., Yamagata, Japan).

### NEP Protein Expression (Immunoprecipitation and Western Blotting)

Rat skin was homogenized and solubilized in RIPA buffer. After immunoprecipitation with a goat polyclonal anti-NEP antibody, western blotting was performed with a mouse monoclonal anti-NEP antibody. Biotinylated anti-mouse IgG was used as a secondary antibody, and then was incubated with streptavidin-horseradish peroxidase conjugate for 1 h. Blots were visualized using ECL detection reagents and chemiluminescence was determined using a Molecular Imager System GS-363 (BIO RAD).

### Northern Blot Analysis for NEP Gene Expression

Total RNAs from rat skin were extracted using ISOGEN and isolated Poly-A RNA by Oligotex-dT30 (Takara, Shiga, Japan). Specific rat NEP primers used were:

Forward, 5′-ATAACAAAATGACATTGGCCAAGC-3′

Reverse, 5′-GTCGTTTTCGACGAAGGTGTATTT-3′

The NEP cDNA sequence is shown in Figure S1, and the primer recognition sites are indicated in bold and underline.

Northern blotting was performed after electrophoresis in 1% agarose gels. After hybridization (68°C, overnight) with the specific probe for rat NEP labeled with ^32^P-dCTP, radioactivity was measured using a BAS2000 bio-image analyzer.

### Immunostaining of NEP in the Rat Hair Cycle

Formalin-fixed paraffin-embedded rat skin sections were blocked with 10% horse serum/PBS for 2 h. Mouse monoclonal anti-NEP antibody (Clone 56C6) was diluted 200 times and placed on each section and then incubated overnight at 4°C. Biotinylated anti-mouse IgG was used as a secondary antibody. VECTASTAIN ABC kit and DAB substrate were used for detection. Hematoxylin was used for counterstaining.

### Elastin Staining in the Rat and Mouse Skin

Staining of elastic fibers in rat and mouse skin was performed by Orcein staining [32].

### Evaluation of Hair Regrowth in Mice

To evaluate hair growth, C3H mice were used with an established method [33], [34] with some modification. After 1 week pre-rearing, approximately 8 cm^2^ of dorsal hair of C3H mouse was shaved with electric clippers, and the skin was treated with a hair removal cream (Kanebo Cosmetics Ltd., Tokyo Japan). Topical application of 0.05% NPLT 100 µl or Vehicle control (80% ethanol) was performed twice per day, 5 days per week starting the day after hair removal. Photographs of the mice were routinely taken during the 28 day test period, and hair regrowth was evaluated by image analysis according to the following formula:
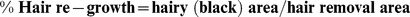
In HE-stained skin sections, growing hair follicles (Anagen III to Anagen VI) were classified according to accepted morphological guidelines [35] and were counted using a microscope. The length of each hair follicle was measured by image analysis using Image J software.

### Human Hair Follicle Organ Culture

Anagen hair follicles were microdissected from normal occipital human scalp skin obtained after written informed consent from two healthy adult males who had undergone routine hair replacement surgery at Skin Clinics (Omiya and Shinjyuku, Japan). This study adhered to the Helsinki Guidelines and was approved by the Institutional Research Ethics Committee of the Kao Corporation (permit Number was 384-20120423). Isolated hair follicles were cultured in Williams’ E medium with glutamine, hydrocortisone, insulin and antibiotics at 37°C in a 95% air, 5% CO_2_ atmosphere. Growing hair follicles were selected one day after isolation, and 0.05% NPLT or vehicle were added to the medium. Digital photographs of cultured follicles were taken using a stereomicroscope every other day and the medium was changed every three days. Lengths of hair shafts were measured by Image analysis using Image J software and the growth rate per day was calculated. When the shape of an anagen hair follicle changed to catagen (dermal papillae separated from the follicular epithelium), we removed the follicle from the length measurement and counted the number of catagen hair follicles.

### Statistical Analysis

All statistical analyses were carried out using SPSS software. One-way ANOVA and post-hoc Tukey’s test, Student’s t-test or Welch’s t-test were used to compare the differences within groups. P<0.05 or 0.01 are considered significant.

## Results

### Fluctuation of ECM-degrading Enzymes during the Hair Cycle

**Figure 1:**
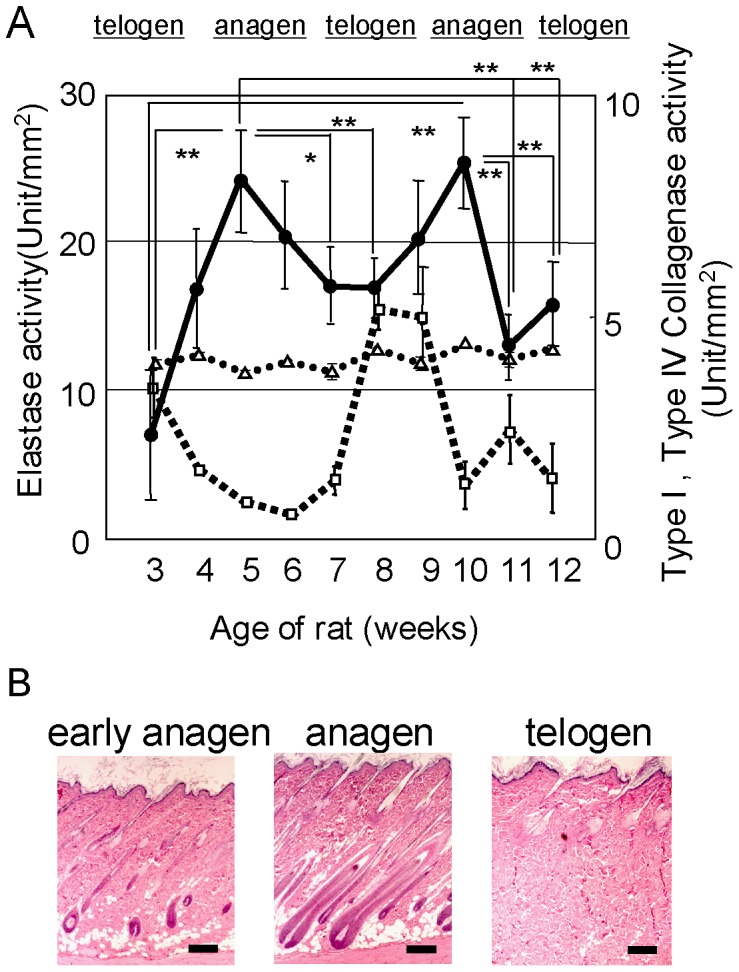
Proteinase activities at each stage of the rat hair cycle. Dorsal skins of SD rats at different hair cycle stages were homogenized and measured for Type I (△) and Type IV (□) collagenases and elastase (•) activities (A). Values represent means ± S.D. from 5 independent experiments. Typical histologic sections of the rat hair cycle (B). Skin specimens (4 mm diameter) biopsied from the dorsal skin at the same time periods used to measure enzyme activities were fixed with formalin and embedded in paraffin. Sections were stained with Hematoxylin and Eosin. Images at early anagen (5 weeks), anagen (6 weeks) and telogen (8 weeks) are shown, as noted. Bar 0.1 mm.

To determine whether ECM-degrading enzymes fluctuate with the hair cycle in skin including the hair follicle, we measured the activities of Type I and Type IV collagenases and elastase as typical ECM-degrading enzymes. We used male SD rats (3 to 12 weeks age) that were 1 week apart in age to study each stage of the hair cycle. The enzyme activities were measured in specific areas of the dorsal skin. Type I collagenase activity was present at almost a steady state throughout the hair cycle (Figure 1A). On the other hand, elastase activity demonstrated large fluctuations with peaks at 5 weeks and 10 weeks of age which correspond to early anagen of the hair cycle (Figure 1A). Type IV collagenase activity demonstrated a fluctuation of activity with a peak at the telogen stage (8 weeks) of the hair cycle (Figure 1A). Tissue sections obtained from contiguous areas at the same times were stained with Hematoxylin and Eosin (HE) after formalin fixation. Typical histological pictures of hair cycle stages are shown in Figure 1B, early anagen (5 weeks), anagen (6 weeks) and telogen (8 weeks).

### Fluctuation of NEP Activity, Protein Expression and Gene Expression Associated with the Hair Cycle

**Figure 2:**
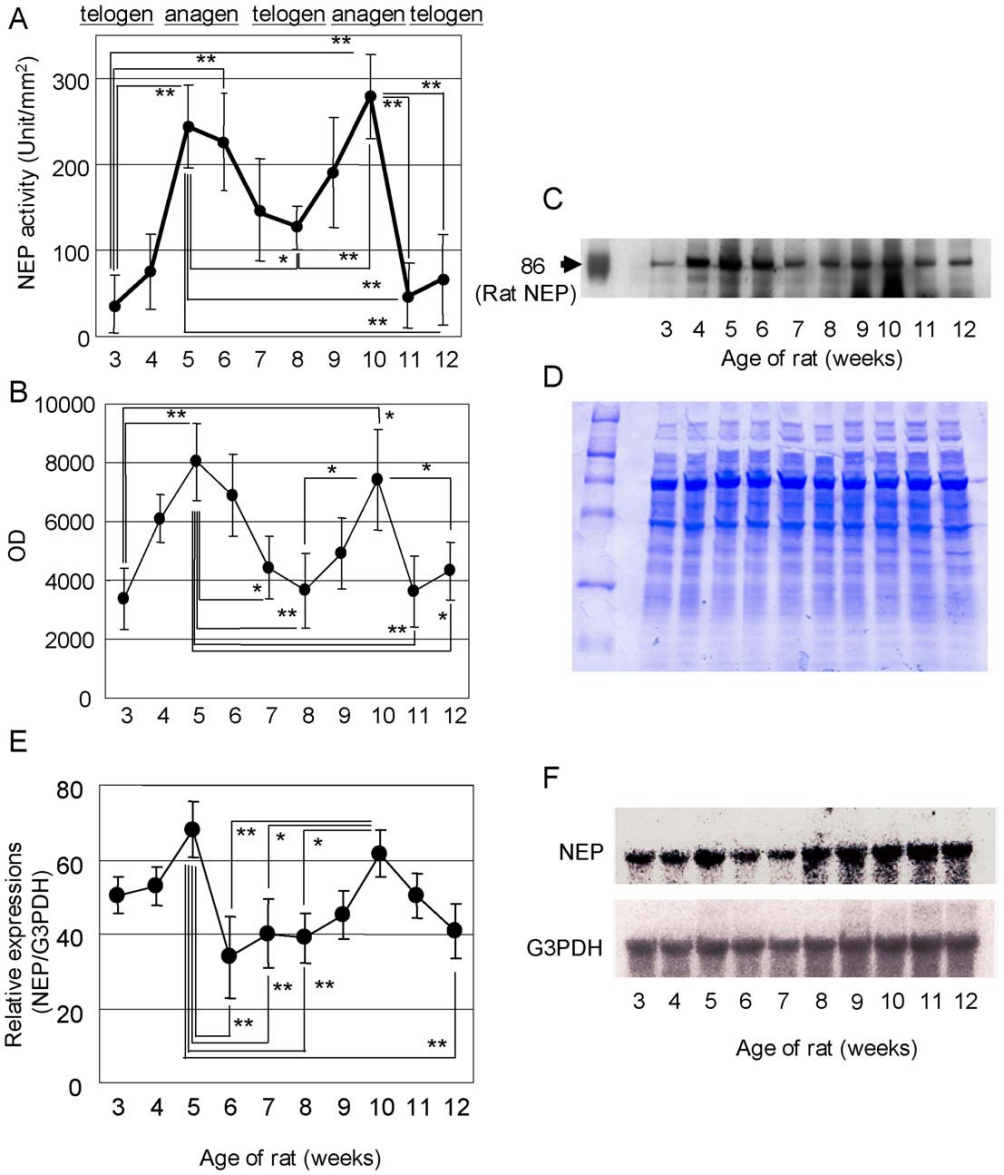
NEP activity and expression at each stage of the rat hair cycle. Dorsal skins of SD rats at different hair cycle stages were homogenized and NEP activity was measured (A). Values represent means ± S.D. from 5 independent experiments. Dorsal skins of SD rats at different hair cycle stages were homogenized and solubilized in RIPA buffer. After immunoprecipitation with an anti-NEP antibody, western blotting was performed. Blots were visualized using ECL detection reagents (C) and chemiluminescence was determined using a Molecular Imager System (B). The loading control is shown by CBB staining (D). Northern blotting was performed with a specific probe for rat NEP labeled with ^32^P-dCTP, radioactivity was measured using a BAS2000 bio-image analyzer (E, F).

Since enzyme activities that are elevated at early anagen are expected to regulate the initiation of the hair cycle, we decided to focus on elastase activity in the skin. Since we demonstrated previously that the major elastase derived from skin fibroblasts is NEP, we next measured NEP activity in the skin of rats including hair follicles at each stage of the hair cycle. As a result, NEP activity also had a very similar pattern of fluctuation to elastase activity, with a peak at early anagen stage (Figure 2A).

Next, we examined whether NEP protein expression fluctuates in a similar manner. Western blotting analysis was performed using rat skin specimens obtained at the same times as used for the enzyme assays. NEP protein expression per area also showed a fluctuation with peaks at 5 and 10 weeks. Thus, the enzyme activity of elastase/NEP seems to be regulated at the protein expression level as revealed by Western blotting (Figure 2B, C). The loading control is shown in Figure 2D.

We further examined whether the fluctuation of NEP protein expression is regulated at the transcriptional level. Northern blotting analysis was performed using mRNA samples extracted from rat skin obtained at same time points as used for the enzyme assays. The relative NEP mRNA level (based on G3PDH) also demonstrated a fluctuation with peaks at 5 weeks and 10 weeks, similar to the peaks in enzyme activity and protein expression. Therefore, NEP expression seems to be regulated at the transcriptional level (Figure 2E, F).

### Localization of NEP in Each Stage of the Hair Cycle

**Figure 3:**
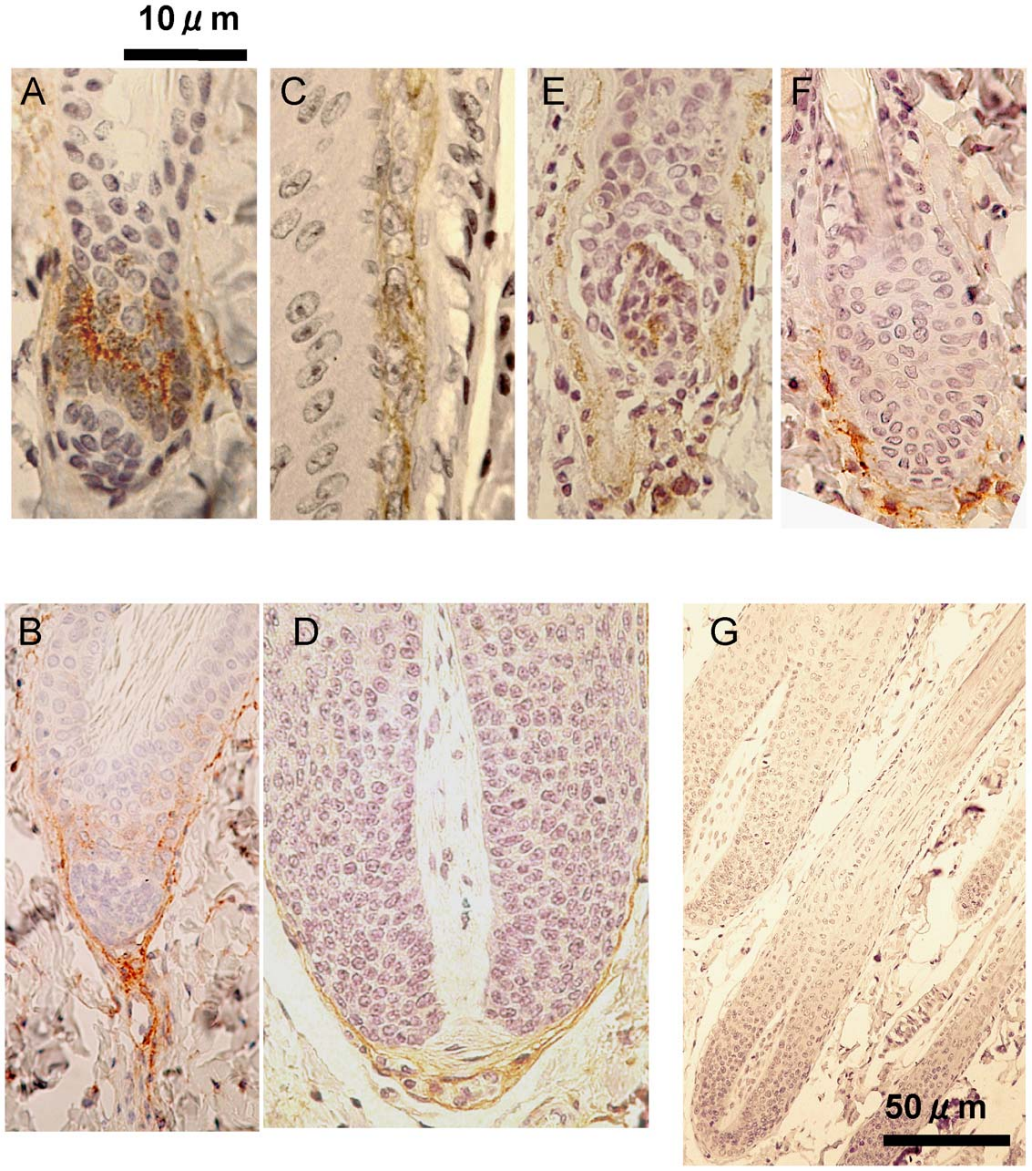
Localization of NEP at each stage of the hair cycle in rats. Immunostaining of rat skin sections including hair follicles was performed with an anti-NEP antibody. In early anagen hair follicles, the follicular epithelium showed strong NEP immunoreactivity (A). Further positive staining existed in the dermis in a direction toward the follicular extension in early anagen (B). Positive staining for NEP appeared at the inner root sheath and the basal plate in anagen (C, D), at the dermal papillae and connective tissue sheath in catagen (E), and weak signals in the fibroblast sheath around club hair were evident in telogen (F). A negative control treated with normal IgG showed no specific staining in any stage of hair cycle or any site of skin (G). Bars = 10 µm (A-F) or 50 µm (G).

To characterize the NEP expression site at each stage of the hair cycle, rat skin specimens obtained at same times as used for the enzyme assays were immunostained with an anti-NEP antibody. In early anagen hair follicles, the follicular epithelium showed strong NEP immunoreactivity (Figure 3A). Further positive staining existed in the dermis in a direction toward the follicular extension in early anagen (Figure 3B). Positive staining for NEP appeared at the inner root sheath and the basal plate in anagen (Figure 3C, D), at the dermal papillae and connective tissue sheath in catagen (Figure 3E), and weak signals at the fibroblast sheath around club hair were evident in telogen (Figure 3F). A negative control treated with normal IgG showed no specific staining in any stage of the hair cycle or any site of skin (Figure 3G).

### NEP Inhibitor Derived from Phosphoramidon

Since NEP activity level and site of expression fluctuated with the hair cycle, we hypothesized that NEP plays a role in regulating the hair cycle. We decided to test that using an NEP inhibitor applied on the dorsal skin of mice. As an inhibitor for NEP, we derivatized phosphoramidon, which is a typical inhibitor for metalloproteinase, to enhance its permeability into the skin and obtained N-phenetylphosphonyl-leucyl-tryptophane (NPLT) [36]. We previously described the synthetic procedure of NPLT and its inability to inhibit type I and type IV collagenase [36]. This indicated that NPLT is a specific inhibitor for NEP. An inhibition curve of NPLT using NEP derived from cultured human fibroblasts shows that it has an IC50 of about 0.008 µM, almost the same level as phosphoramidon (Figure S2A). We demonstrated previously that NPLT has an activity to inhibit fibroblast elastase and has a preventive effect against UV-induced wrinkle formation [36]. We also confirmed the inhibitory activity of NPLT in mouse skin. The topical application of NPLT inhibited skin NEP activity in a concentration-dependent manner, and the concentration over 100 µM revealed maximum inhibitory activity (Figure S2B). Therefore, we used 0.05% (approximately 1 mM) NPLT, which is sufficient to inhibit mouse skin NEP activity, for the in vivo test.

### Effect of the NEP Inhibitor on Regulation of the Hair Cycle in Mice

**Figure 4:**
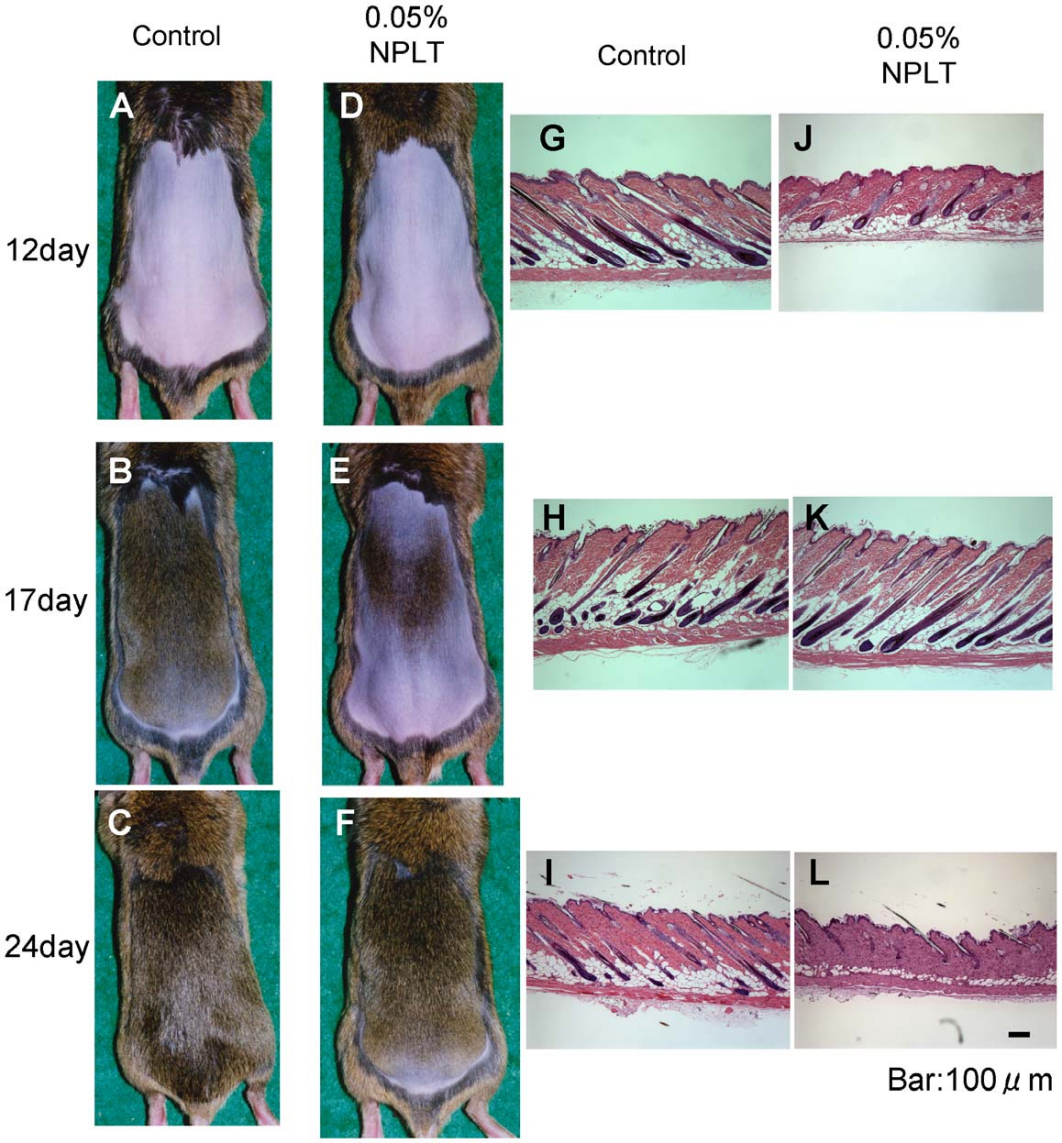
Effect of topical application of the NEP inhibitor on hair re-growth in mice. Eight-week old C3H mice were treated topically with NPLT or with the vehicle control (80% ethanol) twice a day after chemical depilation. Typical photos of dorsal skins (A–F) or HE-stained skin sections (G–L) of C3H mice: left side is vehicle-treated, right side is NPLT-treated mice, upper column 12 days, middle column 17 days, lower column 24 days after depilation.

**Figure 5:**
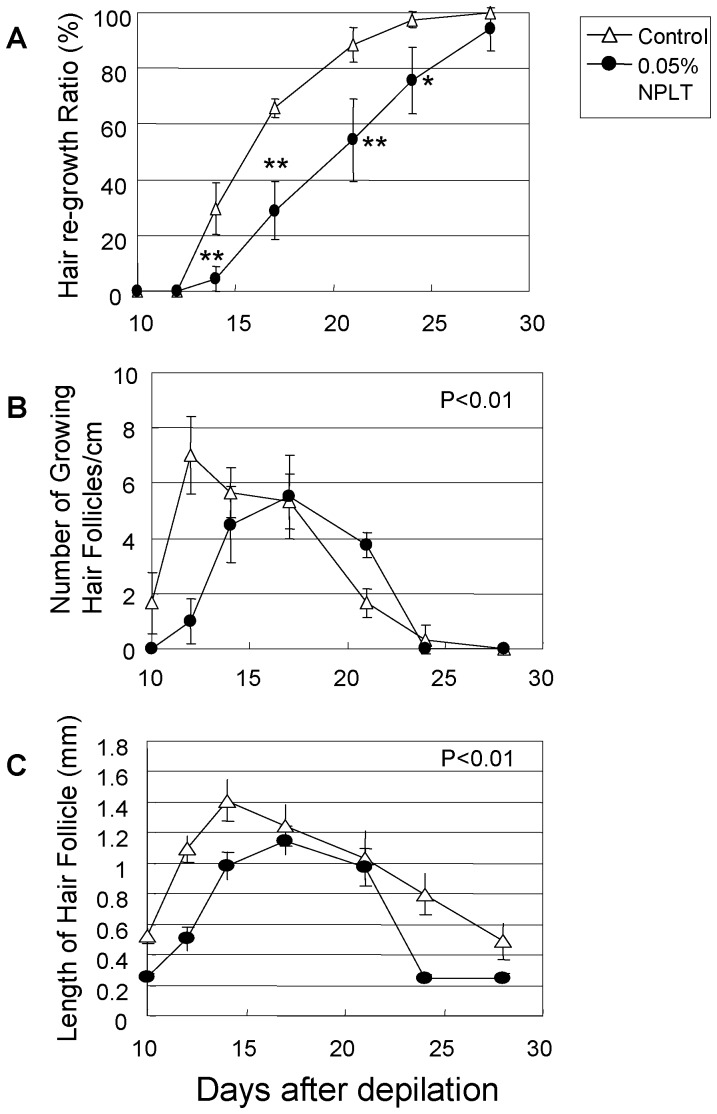
Image analysis of mice hair. Eight-week old C3H mice were treated topically with NPLT or with the vehicle control (80% ethanol) twice a day after chemical depilation. Rate of hair re-growth measured by image analysis at 10, 12, 14, 17, 21, 24 and 28 days after depilation (A). Growing hair follicles (Anagen III to Anagen VI) were classified according to accepted morphological guidelines in HE-stained skin sections, and were counted using a microscope (B). Lengths of hair follicles were measured by image analysis using Image J software (C). NPLT (•) and vehicle control (△). *: P<0.05, **: P<0.01 (vs vehicle control, t test). Data represent means ± SD (n = 5).

To evaluate hair growth, we used C3H mice which have a colored coat, using an established method [33], [34]. Typical photos of the dorsal skin of mice and HE-stained skin section at 0, 17, 24 days after depilation are shown in Figure 4A–L. We show the time course of the rate of hair re-growth after depilation analyzed with photo image analysis in Figure 5A. At 14–24 days after depilation, mice treated with NPLT daily have a significant delay of hair re-growth compared with the vehicle control (Figure 5A). At 24 days after depilation, hairs on the dorsal skin of vehicle-treated mice had recovered almost completely. The complete recovery of NPLT-treated mice was delayed about 1 week. In HE-stained skin sections, the number of growing hair follicles (Anagen III-Anagen VI) were counted (Figure 5B). The day with the maximum number of growing hair follicles was delayed by topical application of 0.05% NPLT. Furthermore, the area under curve (AUC) showed a significant reduction of growing follicles following topical application of NPLT (Figure 5B). The length of hair follicles also showed a delay of hair growth and catagen induction was accelerated by topical application of NPLT (Figure 5C).

### Effect of the NEP Inhibitor on Isolated Human Scalp Hair Follicles in Culture

**Figure 6:**
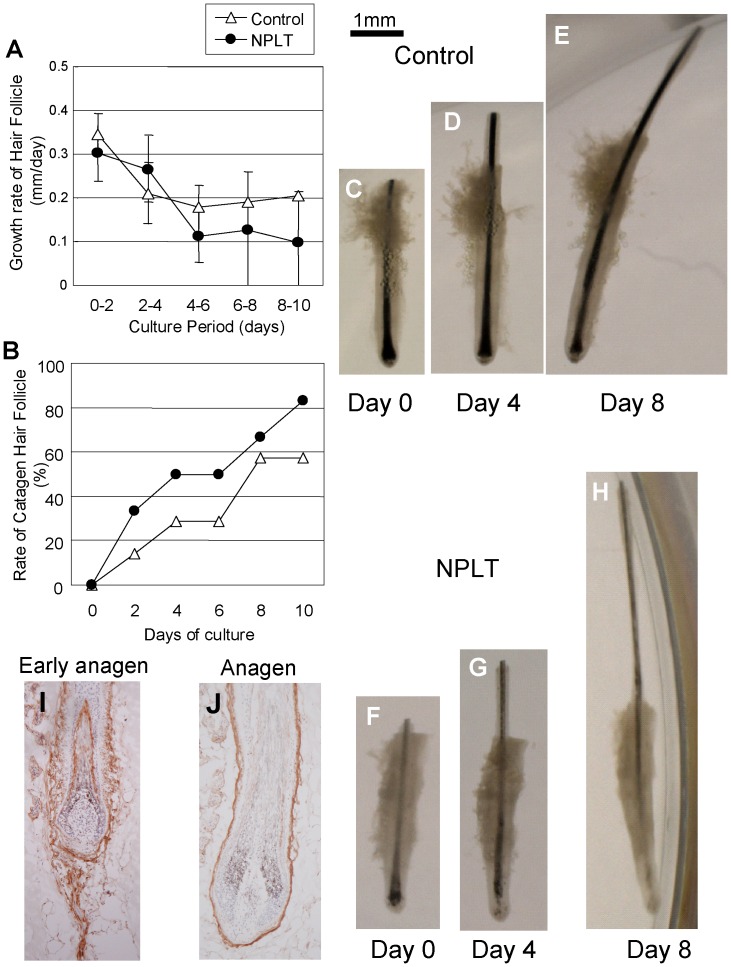
Effect of the NEP inhibitor on human scalp hair follicles in culture. Human hair follicles were isolated from occipital scalp specimens, and were cultivated for 10 days. Lengths of hair shafts were measured by Image analysis using Image J software and the growth rate per day (A) was calculated. Data represent means ± SE (n = 6). When the shape of the anagen hair follicle changed to catagen (dermal papillae separated from the follicular epithelium), the follicle was removed from the length measurement and the number of catagen hair follicles was counted (B). Typical photos of cultured human hair follicles are shown in C–H (C–E: vehicle treated, F–H: NPLT treated). Human scalp skin immunostained with an anti-NEP antibody showed similar positive staining as rat (the follicular epithelium, the dermis intrusive in a direction toward the follicular extension and the connective tissue sheath at early anagen (I), IRS and connective tissue sheath at anagen (J)).

Organ culture of isolated hair follicles should eliminate the influence of extracellular matrix components such as elastin and collagen. Isolated and cultured hair follicles in anagen continue for several days to produce a hair shaft at almost the same speed as occurs in vivo, and enters catagen spontaneously. Growth rates of hair follicles were decreased over time in culture, and at earlier times, there were no differences between NPLT-treated follicles and vehicle-treated follicles (Figure 6A). Concerning the rate of catagen hair follicles, NPLT-treated follicles seem to have accelerated catagen induction (Figure 6B). Typical photos of cultured human hair follicles are shown in Figure 6C–H. Immunohistochemistry of human scalp skin stained with an anti-NEP antibody showed similar positive staining as rat (the follicular epithelium, the dermis in a direction toward the follicular extension and connective tissue sheath at early anagen (Figure 6I), IRS and connective tissue sheath at anagen (Figure 6J)).

### Localization of Elastic Fibers Around Hair Follicles

As previously described [36], the finest oxytalan fibers exist in the upper dermis vertically toward the papillary dermis, while thicker elaunin fibers exist horizontally at the mid-dermis. Around hair follicles, fine elastic fibers exist that surround hair follicles at a higher density than in other areas of the skin (Figure S3A). Elastic fibers further exist in the direction of hair follicles in early anagen (Figure S3C), and at the basal plate and neck of the dermal papilla in mid-anagen (Figure S3B), which is similar to the localization of NEP. Similar observations were reported previously in humans [37], [38]. In NPLT-treated mouse skin (Figure S3E), elastic fibers surrounding early anagen hair bulbs seem to accumulate more than in vehicle-treated mouse skin (Figure S3D).

## Discussion

The hair cycle is a process of cyclic tissue remodeling, and it considered as a complex process that involves many factors, for example, growth factors, cytokines, hormones, adhesion molecules [39] and related enzymes. Here we demonstrate that the expression and activity of a multi-substrate enzyme, NEP, fluctuates according to the hair cycle. We also show that an NEP inhibitor delays the start of the hair growth and accelerates the end of hair growth. These findings strongly suggest that NEP plays some role in the regulation of the hair cycle. Since NEP is known as a multi-substrate enzyme for many neuropeptide and growth factors, some of those substrates might act to regulate the hair cycle. Factors that are substrates of NEP could conceivably regulate the hair cycle negatively, or inactivate the switch to start anagen. For example, an interesting report about galanin, which is one of the neuropeptide substrates of NEP, as an inhibitor of hair growth, was published recently [40]. For another example, Toyoda et al. reported that the expression of substance P (SP), a potent proinflammatory neuropeptide, was enhanced in a patient with alopecia areata (AA), a dermatosis involving the sudden occurrence of bald patches on the scalp, and that NEP (which can degrade SP) is also up-regulated in AA follicles [24]. They speculated that the up-regulation of NEP reduces the amount of SP to limit the proinflammatory effect. Arck et al. also demonstrated that SP is involved in stress-induced catagen [41]. These data support the hypothesis that NEP inhibits catagen induction by degrading an inflammatory neuropeptide such as SP which then induces catagen.

Previously we determined that NEP has an elastin degrading activity [18]. It wouldn’t be a surprise if elastin, which is a component of the ECM, has some involvement in hair cycle control, because the hair cycle is considered as a kind of process of tissue remodeling. In fact, histochemical images showed that elastic fibers exist that surround hair follicles at a higher density than in other areas of the skin [37]. Another study reported that an elastin-like body, called the “Arao-Perkins body”, which exists in the neck of the dermal papilla, forms anew every hair cycle and is stuck in the follicle. In male pattern alopecia, the elastin-like bodies accumulate in collapsed fibrous root sheaths and fill in hair follicle spaces like a ladder [38]. This may prevent new hair from growing down deeply, and the hair would become shorter than its predecessor as a consequence. This suggests the necessity for elastin degradation to expand space for growing new hair.

Divano et al. reported that levels of collagen and elastin fluctuate in association with the hair cycle [42]. They reported that collagen remains unchanged during anagen, while elastin content gradually increases throughout anagen and decreases in telogen. These data can be explained by our findings about the activities of collagenase and elastase that mediate the degradation of ECM components. They also suggested the possibility that growing hair follicles provoke the expansion of the ECM, which can block the downwards movement of previously growing follicles into the dermis or the readjustment of the skin after expansion by growing follicles. In our study, we observed that the localization of NEP changes depending on the phase of the hair cycle. These results are consistent with a previous report [20], [21]. The change in the localization of NEP also suggests the possibility that NEP plays some role in regulating the hair cycle.

NEP is also known as CD10 and CALLA and is expressed in many types of tumors [43]. NEP was reported to play some role in the metastasis of gastric carcinomas [44] and of colon tumors to the liver [45]. It is well known that degradation of the ECM is an important step in the metastasis of a tumor. The invasion of tumor cells into target organs might be similar to the extension of early anagen hair follicles into the skin, because both processes need to break down the ECM around the tissues.

We used three species of animals in this study, rats for enzyme activity, protein and mRNA expression, mice for the inhibitor effects, and human hair follicles for organ culture. We have two reasons for this. One is for convenience, that is, the dorsal skin of rats is large enough to simultaneously measure NEP activity, elastase activity, as well as the expression of NEP protein and mRNA. On the other hand, since the dorsal skin of mice is smaller, we need to apply only a smaller amount of inhibitor. Further, mouse skin has an easy and well established method to evaluate hair re-growth. Human hair follicles are easy to isolate and culture, although it is hard to obtain each stage of the hair cycle. The second reason is to indicate that the role of NEP in regulating the hair cycle is not a species specific event. The localization of NEP and elastin around hair follicles as reported previously in humans is similar to our data in rats and mice. Since the substrate elastin is rich around hair follicles, NEP, which is up-regulated in epithelial cells and in connective tissue sheaths at early anagen to anagen, suggests that one of the targets is elastin. We speculated that NEP fluctuation is associated with the hair cycle and that its regulatory role in the hair cycle through the degradation of elastin occurs universally beyond the difference between species. The role of NEP which appears in the dermal papilla at catagen is still unclear, but the dermal papilla, which is surrounded by epithelial cells during anagen, becomes uncoupled in catagen and is exposed to many biological factors outside the follicle. One possible role of NEP is to protect the dermal papilla from those factors, such as SP, by enzymatic degradation. The role of NEP throughout the hair cycle needs further investigation.

Our data suggest at least one important role of NEP in regulating the hair cycle. This finding has the potential to be used in therapeutic methods to control hair growth, for example, to develop a treatment method for unwanted hair growth.

## Supporting Information

Figure S1 **Nucleotide sequence of rat NEP cDNA with primer recognition sites indicated in boldface and underline.** ACCESSION: NM_012608 DEFINITION: Rattus norvegicus membrane metallo-endopeptidase (Mme), mRNA
(TIF)

Figure S2 **Inhibitory Effect of NPLT on NEP activity.** Concentration-dependent inhibition of NEP by NPLT and by phosphoramidon (A). Inhibition curve of NPLT (•) using NEP derived from cultured human fibroblasts shows an IC50 of about 0.008 µM, almost the same level as phosphoramidon (○). NEP activity in mouse skin was measured 1 day after topical application of NPLT at the indicated concentration (B). Values represent means ± S.D. from 3 independent experiments.
(TIF)

Figure S3 **Localization of elastic fibers in rat and mouse skin around hair follicles.** Orcein staining visualizing elastin as dark brown fibers. Finest oxytalan fibers exist in the upper dermis vertically toward the papillary dermis, and thicker elaunin fibers exist horizontally at mid dermis. Around hair follicles, fine elastic fibers exist that surround hair follicles at a higher density than in other areas of the skin (A). Elastic fibers further exist at intrusive direction of hair follicle in early anagen (B), and at the basal plate and the neck of dermal papilla in mid-anagen (C) similar to the localization of NEP (rat). In NPLT-treated mouse skin (E), elastic fibers surrounding an early anagen hair bulb seems to accumulate more than in the vehicle-treated mouse skin (D). Bar = 100 µm (A–C), 10 µm (D, E)
(TIF)
