# Supplementary figures and images for: The Role of Neprilysin in Regulating the Hair Cycle
Source: PLoS One. 2013 Feb 13;8(2):e55947. doi: 10.1371/journal.pone.0055947 (PMC3572137; doi:10.1371/journal.pone.0055947)

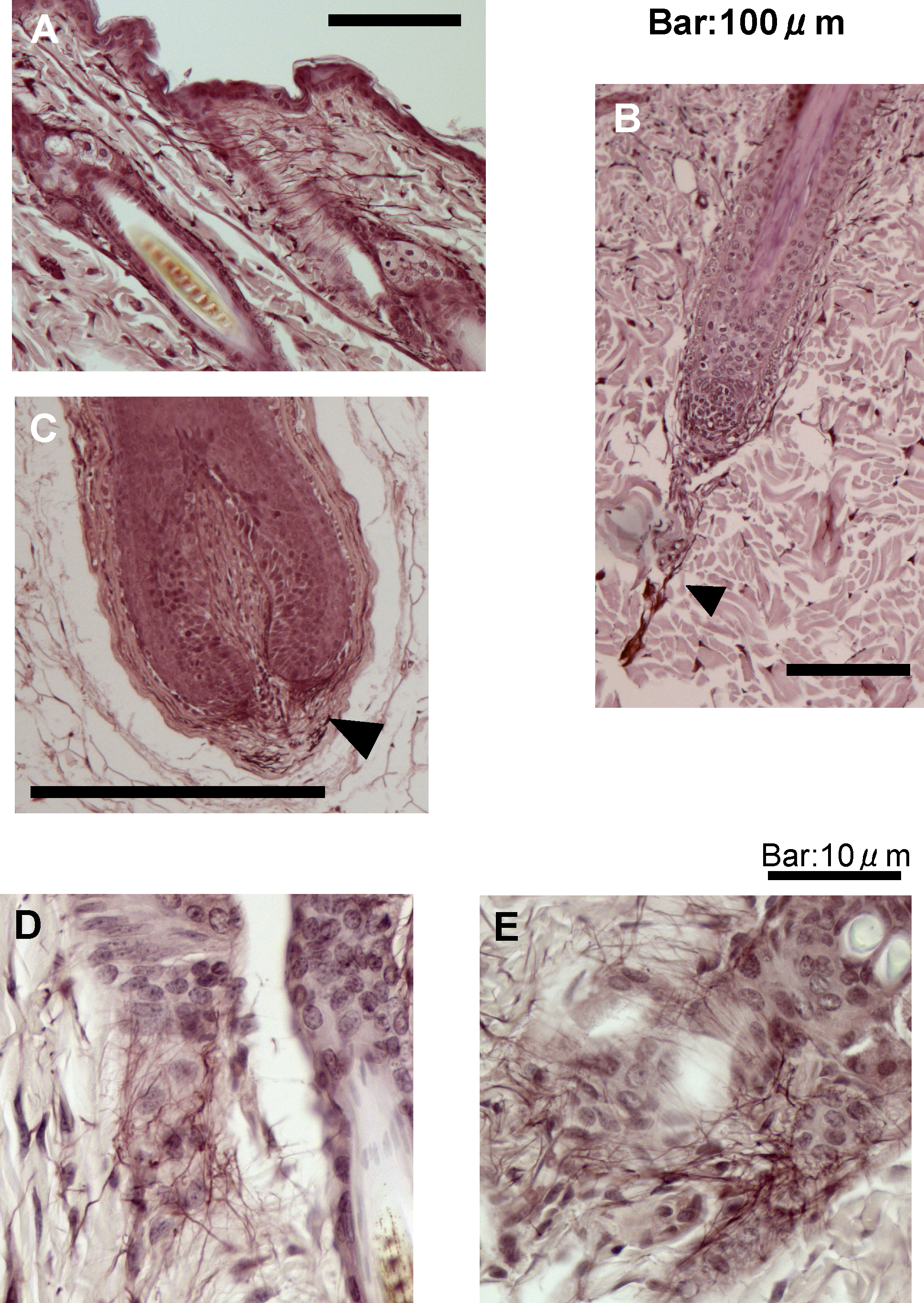

Supplement: Figure S3 — Localization of elastic fibers in rat and mouse skin around hair follicles. Orcein staining visualizing elastin as dark brown fibers. Finest oxytalan fibers exist in the upper dermis vertically toward the papillary dermis, and thicker elaunin fibers exist horizontally at mid dermis. Around hair follicles, fine elastic fibers exist that surround hair follicles at a higher density than in other areas of the skin (A). Elastic fibers further exist at intrusive direction of hair follicle in early anagen (B), and at the basal plate and the neck of dermal papilla in mid-anagen (C) similar to the localization of NEP (rat). In NPLT-treated mouse skin (E), elastic fibers surrounding an early anagen hair bulb seems to accumulate more than in the vehicle-treated mouse skin (D). Bar = 100 µm (A–C), 10 µm (D, E) (TIF) [file pone.0055947.s003.tif]
